# Prognostic and Predictive Roles of *KRAS* Mutation in Colorectal Cancer

**DOI:** 10.3390/ijms131012153

**Published:** 2012-09-25

**Authors:** Amanda K. Arrington, Eileen L. Heinrich, Wendy Lee, Marjun Duldulao, Supriya Patel, Julian Sanchez, Julio Garcia-Aguilar, Joseph Kim

**Affiliations:** 1 Division of Surgical Oncology, City of Hope Comprehensive Cancer Center, Duarte, CA 91010, USA; E-Mails: eheinrich@coh.org (E.L.H.); wenlee133@gmail.com (W.L.); philodee050382@yahoo.com (M.D.); jusanchez@coh.org (J.S.); 2 Department of Surgery, University of Southern California, Los Angeles, CA 90001, USA; E-Mail: supriya.patel@gmail.com; 3 Division of Colorectal Surgery, Memorial Sloan-Kettering Cancer Center, New York, NY 11597, USA; E-Mail: garciaaj@mskcc.org

**Keywords:** *KRAS*, colorectal cancer, colon cancer, EGFR, Let7, ras, oncogenes

## Abstract

The *RAS* gene family is among the most studied and best characterized of the known cancer-related genes. Of the three human ras isoforms, *KRAS* is the most frequently altered gene, with mutations occurring in 17%–25% of all cancers. In particular, approximately 30%–40% of colon cancers harbor a *KRAS* mutation. *KRAS* mutations in colon cancers have been associated with poorer survival and increased tumor aggressiveness. Additionally, *KRAS* mutations in colorectal cancer lead to resistance to select treatment strategies. In this review we examine the history of *KRAS*, its prognostic value in patients with colorectal cancer, and evidence supporting its predictive value in determining appropriate therapies for patients with colorectal cancer.

## 1. Introduction

The *RAS* oncogene has a well established role in cell growth and regulation; and its protein product affects many cellular functions including cell proliferation, apoptosis, migration, fate specification, and differentiation. There are three known human isoforms, *NRAS*, *HRAS*, and *KRAS*. Over 90% of pancreatic adenocarcinomas [1], 30%–50% of colorectal cancers [2–4], 55% of thyroid cancers [5], 35% of lung cancers [5], and 35% of rhabdomyosarcomas [6] harbor mutated *RAS* genes. Although *HRAS* was historically the most studied *RAS* gene, it is actually the isoform least mutated in human cancers [7]. In fact, *KRAS* mutations comprise 86% of all *RAS* mutations [8]. Mutations in *KRAS* occur with the greatest frequency in all human cancers (21.6%), followed by *NRAS* (8.0%), and *HRAS* (3.3%) [8]. *KRAS* was initially identified in a human lung cancer cell in 1982 and, since then has been shown to be mutated in 35%–50% of all non-small cell lung cancers [9]. Although a common mutation in cancer, *KRAS* has been difficult to therapeutically target. A better understanding of this gene, as well as its interactions with other genes and mutations, has recently revealed its potential prognostic and predictive roles in tumor aggressiveness and patient outcomes. In this paper we highlight the current state of understanding of *KRAS*, with a specific emphasis on the role of *KRAS* in colorectal cancer.

## 2. History of *KRAS*

*RAS* is the name given to a family of related genes that encode a class of 21 kD membrane-bound proteins that bind guanine nucleotides and have intrinsic GTPase activity. The first two *RAS* genes, *HRAS* and *KRAS*, were identified in 1975 from studies of two cancer-causing viruses, the Harvey sarcoma virus and Kirsten sarcoma virus, by Scolnick *et al.* at the National Institutes of Health (NIH) [10]. The human analog of this gene was subsequently discovered in 1982 and has been intensely studied and implicated in the pathogenesis of many cancers. Of the three known human *RAS* genes, *KRAS* is most frequently mutated in cancer [7].

**Figure 1:**
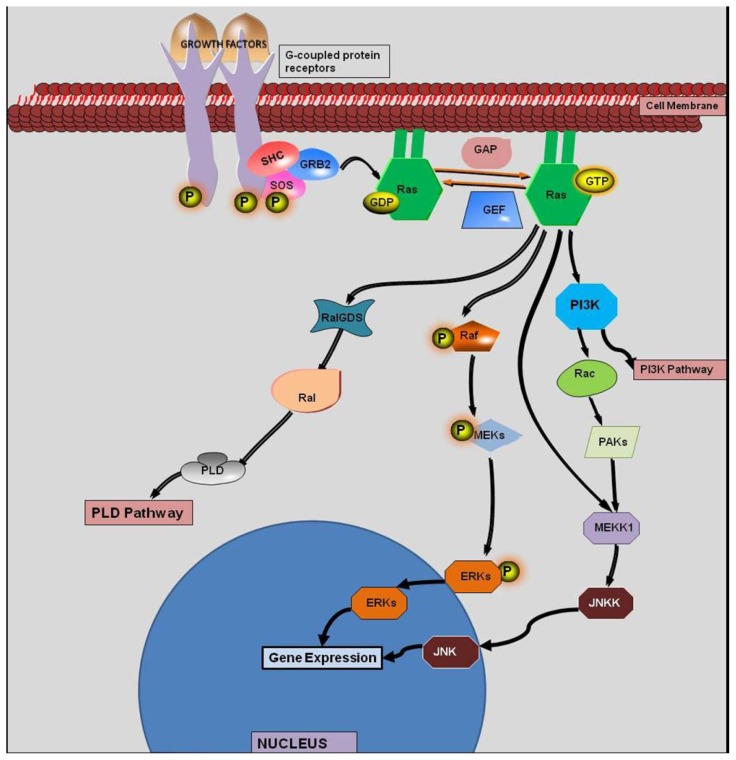
The *Ras* Activation Cascade.

**Figure 2:**
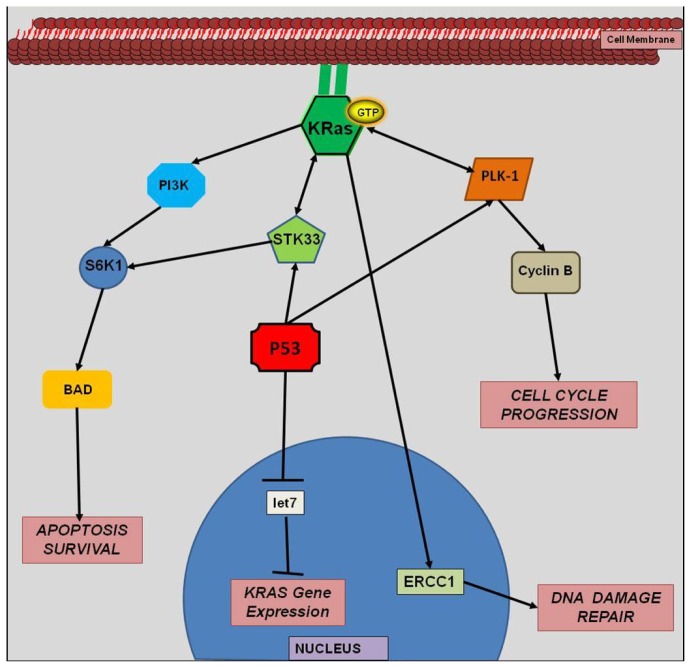
Constitutional Activation of *KRAS*.

The *KRAS* gene encodes a 188 amino acid protein that has inherent catalytic activity. Post-translation modification of this protein facilitates its localization to the cell membrane. Normally, ras proteins exist in an inactive state in any given cell. All members of the ras family become activated when a nearby transmembrane receptor (e.g., growth factor receptors, G-protein coupled receptors, toll-like receptors, *etc*.) is bound by its corresponding ligand (Figure 1). The subsequent intracellular signal cascade involves guanine exchange factors (GEF) which facilitate the activation of ras by replacing the inactive GDP with GTP. Once activated, ras leads to the downstream activation of a wide variety of effectors including serine/threonine kinases, GTPase-activating proteins (GAPs), phosphoinositide 3-kinase (PI3K), and GEFs [11]. Ras is deactivated when the GTP molecule is converted back to a GDP molecule [11]. If *KRAS* is mutated, it remains in the GTP state. Therefore, *KRAS* remains in a constitutive GTP-bound state and, thus, regulation of downstream functions is lost (Figure 2). For example, the dysregulated GTP-bound activation of mutant-derived *KRAS* protein leads to unregulated downstream cell-growth.

A number of reports have investigated the significance of specific *KRAS* point mutations which typically involve codons 12, 13, 59 or 61 [12,13]. Nearly 97% of all *KRAS* mutations are localized to codons 12 or 13. As shown in Table 1, point mutations in codon 12 are the most common *KRAS* mutation in colorectal cancer [14–17]. The most frequent change is the transition of GGT to GAT in codon 12 [18]. Specifically, DNA nucleotide mutations involving G→A or G→T, lead to a change in the glycine amino acid in the first or second location. The presence of a glycine residue in codon 12 appears to be critical for the normal function of ras proteins [19,20]. Therefore, single base substitutions that result in the replacement of the glycine amino acid with another amino acid in this location result in the formation of GTPases that are locked in the “on” position.

## 3. *KRAS* and Prognosis in Colorectal Cancer

*KRAS* is perhaps best characterized in colorectal cancer. In 1988, Vogelstein *et al.* first proposed a model for a sequence of genetic events leading to the development of colorectal cancer [4]. In this model, point mutations in *KRAS* were described as an early event in the pathogenesis of colorectal cancer. In fact, *KRAS* mutations were demonstrated in 50% of adenomas and described as a key genetic alteration necessary for the progression of adenoma to colorectal cancer. Thus, many have hypothesized that development of *KRAS* mutation is an important role in the multi-step process early in carcinogenesis. Since only 30%–50% of colorectal cancers have *KRAS* mutations [4,18,21], there has been speculation that the detection of *KRAS* mutation may portend a worse prognosis. Unfortunately, the reports have been contradictory on the prognostic value of *KRAS* mutations [15,22–30]. There have been discrepancies in these reports because of inconsistencies in defining prognosis. For example, Tanaka *et al.* reported that *KRAS* mutation was an independent factor associated with prognosis in a multivariate analysis [23], whereas Dix *et al.* reported that *KRAS* mutation was not prognostic in their cohort when predicting short-term survival [27]. Therefore, the primary goal of The Kirsten Ras In-Colorectal-Cancer Collaborative Group (RASCAL) was to definitively determine whether the presence of a *KRAS* mutation is of prognostic significance. Andreyev *et al.* clarified in the RASCAL study the association of specific *KRAS* mutations with patient outcomes and tumor characteristics [17,22]. This study included primary data from 2721 colorectal cancer patients from 22 research groups in 13 different countries [18]. In their multivariate analyses, the presence of *KRAS* mutation was significantly associated with poorer prognosis [18].

The secondary goal of the RASCAL study was to determine whether select point mutations had prognostic significance. A codon 12 mutation was recorded in 755 (27.7%) patients and codon 13 mutation was identified in 146 patients (6.6%) [18] (Table 1). Of the 723 patients with identified and isolated codon 12 or 13 *KRAS* mutation, the most common alteration was glycine to aspartate substitution on codon 12 (30.6%; *n* = 221 of 723), whereas the next most common mutation was glycine to valine on codon 12 (23.4%; *n* = 169 of 723) [18] (Table 1). There was no difference in the rate of *KRAS* mutation among different histologic stage, tumor site, gender, geographic location, or age. However, G→A transitions were found more frequently in patients with an anastomotic recurrence (58.2%) than in patients with other types of recurrence [18]. However, progression-free survival was lower and risk of death was increased with the detection of any *KRAS* mutation [18]. When comparing all of the specific point mutations (Table 1), overall survival was adversely affected by the presence of a glycine to valine amino acid substitution on codon 12 (*i.e.*, C12V) in the RASCAL study [18,21]. Additionally, any G to T transition on codon 12 (which lead to a valine or cysteine amino acid substitution) was an independent marker of both disease-free survival and overall survival. *KRAS* C12V mutation was the most significantly associated with an adverse outcome [18]. A recent study of 201 patients with advanced colorectal cancer further illustrated a significant difference in progression free survival between patients with codon 12 and 13 mutant tumors treated with chemotherapy [31].

Similarly, in a study of 392 primary colorectal cancer patients in a single institution, Zlobec *et al.* reported a *KRAS* mutation in 30.1% of cases [32]. In a univariate analysis, patients with *KRAS* G12D mutation had poorer prognosis compared to all other patients. Furthermore, the detection of G12D had a significant adverse effect on outcomes when compared to patients with other *KRAS* mutations [32].

Andreyev *et al.* subsequently investigated a larger patient population in the RASCAL II study with the goal of evaluating the impact of *KRAS* mutations on different stages of colorectal cancer [21]. RASCAL II confirmed that a glycine to valine mutation on codon 12 of the *KRAS* gene has a significant association with biological behavior of colorectal cancer. In particular, this specific mutation was associated with a 50% increased risk of relapse or death in patients with Dukes’ stage C cancer. However, the authors were unable to correlate a similar increased risk in patients with Dukes’ Stage B cancer [21].

*KRAS* mutations have also been associated with more rapid and aggressive metastatic behavior of colorectal liver metastases. Nash *et al.* observed that both *KRAS* mutation and high KI-67 expression were associated with multiple liver metastases, shorter time interval to their detection; and with poor survival after colon resection [33]. Additionally, *KRAS* mutation was independently associated with poor survival after liver resection [33]. Santini *et al.* also showed that *KRAS* C12V mutations were more frequently associated with hepatic metastasis [34]. Further, in a recent study of 143 Korean patients with metastatic or recurrent colorectal cancer, lung metastasis was more frequently the initial metastatic site in patients with the KRAS mutations [35].

## 4. *KRAS* Mutations and Response to Therapy

### 4.1. *KRAS* and EGFR

**Figure 3:**
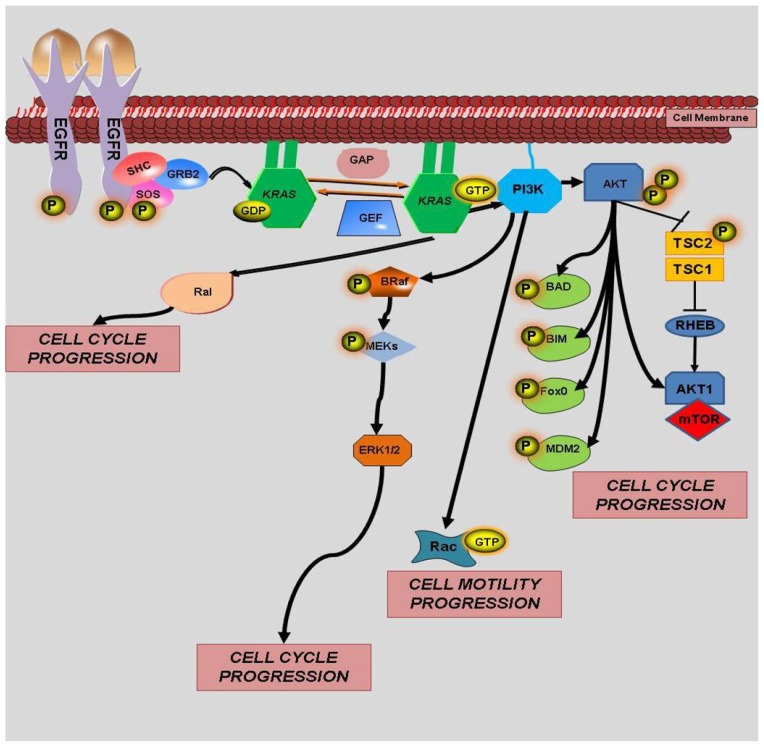
EGFR Signaling and *KRAS*/*BRAF* Interaction.

As expected, an improved response to therapy correlates with more favorable outcomes. In metastatic colorectal cancer, increased response rates to treatment are associated with improvements in progression free survival and overall survival [36]. However, the detection of *KRAS* mutations has been associated with decreased response rates to select chemotherapeutic agents. Therefore, *KRAS* mutational status is a critical factor when considering the use of targeted therapies. The association of *KRAS* gene mutation and response to therapy was first reported in patients with metastatic colorectal cancer, who were treated with anti-epidermal growth factor receptor (EGFR) agents. Lievre *et al.* first reported the link between *KRAS* gene mutation and decreased response to anti-EGFR agents [37]. Additionally, they noted in a retrospective analysis that patients with wild-type *KRAS* had better overall survival compared to patients harboring mutant *KRAS* [37]. Given that EGFR, a transmembrane receptor tyrosine kinase, is overexpressed in 25%–75% of colorectal tumors [38,39], a significant proportion of patients that may benefit from anti-EGFR agents may also have *KRAS* mutation. Similar to *KRAS* mutations, EGFR overexpression has also been linked to poor prognosis and increased risk of metastasis in colorectal cancer and, therefore, represents a promising therapeutic target [40,41]. Upon activation of EGFR, K-RAS is activated downstream [42] and results in downstream signaling through the PI3K and extracellular signal regulated kinase (ERK) pathways [42] (Figures 2 and 3). Thus, K-Ras is a critical mediator of EGFR-induced signaling cascades (Figure 3). Accordingly, other alterations in the mediators of the EGFR pathway (e.g., mutation of *BRAF*, *PTEN*, *PIK3CA*, *etc*.) could further modify the response to anti-EGFR therapies [43–46].

Numerous EGFR blockers have been investigated to date. Unfortunately, resistance to anti-EGFR therapies has been observed in patients with *KRAS* mutation. The recent OPUS and CRYSTAL trials examined the addition of an anti-EGFR monoclonal antibody cetuximab, to first-line FOLFOX (leucovorin, 5-flurouracil [5-FU], oxaliplatin) or FOLFIRI (leucovorin, 5-FU, irinotecan) chemotherapy and illustrated that the addition of anti-EGFR agents did not improve response in patients with mutant *KRAS* and may, in fact, be detrimental [43–46]. Two other recent large randomized prospective studies have further demonstrated the effect of *KRAS* mutation on response to anti-EGFR monoclonal antibodies cetuximab or panitumumab [47,48].

Not all specific point mutations in *KRAS* may underlie the resistance to anti-EGFR monoclonal antibodies. De Roock *et al.* demonstrated in a pooled analysis of 579 patients compiled from seven clinical trials examining metastatic colorectal cancer patients, that patients overall with *KRAS* mutation had decreased survival but those with *KRAS* G13D mutation had better overall survival and progression-free survival after treatment with cetuximab compared to other *KRAS* mutant tumors [49]. Conversely, *KRAS* G13D mutation has also been associated with worse overall survival compared to patients with other *KRAS* mutations or wild-type *KRAS* [49,50]. Interestingly, Tejpar *et al.* reported in a pooled analysis of 533 metastatic colorectal cancer patients from the CRYSTAL and OPUS trials that patients with *KRAS* G13D mutation had a poorer response to first-line chemotherapy compared to other *KRAS* mutations and wild-type *KRAS*. However, they also noted that the addition of cetuximab to first-line chemotherapy may benefit patients with *KRAS* G13D mutation [51,52].

### 4.2. *KRAS* and *BRAF*

Several retrospective studies have indicated that the mutation status of *BRAF* may also be predictive of response to anti-EGFR therapies in patients with metastatic colorectal cancer [49,53,54]. Downstream from Ras, there are 3 different Raf kinases: A-Raf, B-Raf and C-Raf. The best characterized and studied in tumorigenesis is B-Raf. In sporadic colorectal cancers, up to 15.7% of colorectal cancers have a *BRAF* mutation [55]. After binding and activation by GTP, ras recruits B-Raf; this phosphorylates ERK, thereby initiating ERK/MAPK signaling leading to gene expression (Figure 3). Interestingly, *BRAF* mutations, which are predominantly mutually exclusive of mutant *KRAS*, have also been associated with resistance to anti-EGFR treatment in colorectal cancer [53,55]. In the OPUS study, *BRAF* mutations were detected in only 4% (11 of 309) of the tumor specimens and all of these specimens harbored wild-type *KRAS*. Given the small sample size, no definitive conclusions could be reached about prognosis [43,46]. Similarly, 6% (60 of 999) of tumor samples in the CRYSTAL study had a *BRAF* mutation, mainly in the setting of wild-type *KRAS* [44,45]. Interestingly, 1 patient had *KRAS* mutation and *BRAF* mutation; and, therefore, the effect of both mutations could not be assessed. The presence of *BRAF* mutation in this study correlated with poor prognosis and worse outcomes when compared to tumors with wild-type *BRAF* [44,45].

### 4.3. *KRAS* Mutations in Locally Advanced Rectal Cancer

Radiation therapy is a mainstay in the management of patients with rectal cancer. It is well established that more than 2/3 of patients derive some form of tumor downstaging with radiation therapy [56,57]. Furthermore, up to 25% of these patients may have complete eradication of their tumors or achieve a pathologic complete response (pCR) [58]. *KRAS* mutation status appears to influence response to therapy in patients with locally advanced primary rectal cancer. In a retrospective study examining 146 patients with locally advanced rectal cancer, Bengala *et al.* demonstrated that patients with mutated *KRAS* had a decreased rate of complete response to concomitant chemoradiation with continuous infusion of 5-FU with or without oxaliplatin or capecitabine compared to wild-type patients (7.4% *vs*. 19.2%) [59]. However, the overall rate of complete response was low (14.4%) and the difference in rate of complete response was not found to be significant between *KRAS* wild-type and mutant patients. In our own prospective multicenter study of a 132 rectal cancer patients treated with neoadjuvant chemoradiation therapy (NCRT), we determined that *KRAS* mutations were more common in non-pathologic complete response (non-pCR) patients compared to patients with a pCR (49% *vs*. 24%, *p* = 0.014) [60].

### 4.4. miRNA and *KRAS* Interactions

*KRAS* mutations may prove both predictive of treatment efficacy and prognostic for patient outcomes; however, effective and optimized *KRAS* silencing therapeutic strategies remain to be developed [61]. The prevalence of *KRAS* mutations in a variety of cancers underscores the promise that targeting *KRAS* may have in treating cancer. Recent studies suggest that use of microRNAs (miRNA) which target *KRAS* may be a way to stop the aberrant activation of this protein.

miRNAs are 20–25 nucleotide long sequences of endogenous non-coding RNA which bind to the 3′-untranslated regions (UTRs) of their corresponding target messenger RNAs (mRNAs) to regulate gene expression [62,63]. miRNAs block transcription if they show perfect complementarity to the mRNA target, or block translation if there is partial complementarity [64]. In the search for novel strategies in the treatment of colon cancer, miRNAs have received attention for their role in controlling gene expression [65]. The complexity of miRNA regulation of gene expression lies in the ability of one single miRNA to have oncogenic or tumor-suppressive effects on more than one pathway [66]. In addition, miRNA expression is often lost in cancer because they tend to be located in genomic regions that are frequently lost during cancer development [67]. Some of the better understood miRNAs that play a role in cell proliferation in colorectal cancer are lethal-7 (let-7) and miR-143 [64,66,68,69]. These deregulated miRNAs have been associated with cell proliferation and have been investigated for their interactions with *KRAS* [66].

### 4.5. let-7 miRNA and *KRAS*

Several studies have demonstrated that the family of let-7 miRNAs is an important regulator of *KRAS* [66,70] (Figure 2). The family of *RAS* genes has several let-7 complementary sites at the 3′-UTR, enabling let-7 miRNA to bind to and regulate *KRAS* gene expression [70]. The reduced expression of let-7 in cancer tissues corresponds to significantly higher levels of KRAS mRNA [70]. Johnson *et al.* reported that ras protein levels were decreased up to 70% in both hepatocellular carcinoma cells and cervical cancer cells treated with let-7 miRNA [70]. In addition to the important mechanistic role of let-7 miRNA in post-transcriptional regulation of *KRAS* gene expression, decreased let-7 miRNA expression has been associated with worse patient outcomes in lung cancer [71] and may also affect radiosensitivity. In a lung cancer model, Jeong *et al.* reported that the overexpression of let-7 miRNA inhibited translation of *KRAS* and subsequently increased the sensitivity of cells to ionizing radiation [72].

There is growing focus on elucidating the role of let-7 miRNA in colon cancer, since it may be a *KRAS*-driven cancer. Akao *et al.* recently showed that let-7 miRNA may have a suppressive effect on growth and proliferation in human colon cancer cells [73]. In DLD-1 human colon cancer cells, which have low endogenous levels of let-7, transfection with the let-7a precursor miRNA significantly abrogated growth potential and concomitantly decreased the levels of ras and c-MYC proteins [73]. In an analysis of human non-metastatic colon cancers, expression of an upstream repressor of let-7, *LIN28B*, was associated with poorer patient survival [74,75]. Using an *in vivo* model, King *et al.* also showed that *LIN28B+* tumors, which have repressed let-7, have increased metastases and stem cell markers that can sustain cancerous growth [74].

It has been shown that a T→G single nucleotide polymorphism (SNP) in the let-7 complementarity site on the *KRAS* UTR causes decreased binding of let-7 to *KRAS* which in turn leads to increased *KRAS* expression. In metastatic colon cancer patients harboring a *KRAS* mutation, the presence of this variant led to decreased progression-free and as well as decreased overall survival [76]. Further research is required to determine more precisely how let-7 is regulated and if its expression or the expression of its repressor(s) can be used to target *KRAS*-driven cancers.

### 4.6. miR-143 and *KRAS*

miR-143 is one of the most downregulated miRNAs in colorectal cancer samples compared to normal adjacent tissue specimens [69,77]. Like let-7 miRNA, miR-143 has been implicated in colorectal cancer as a link to KRAS-driven carcinogenesis [64,68,69,77,78] and is shown to bind to the 3′-UTR of the *KRAS* gene [77]. The role of miR-143 as a tumor suppressor was demonstrated through the ability of miR-143 inhibition to increase KRAS protein levels and cell proliferation *in vitro* [77]. Conversely, treating cells with a miR-143 mimic or overexpressing miR-143 in colorectal cancer cells, knocked down expression of *KRAS*, decreased activation of ERK1/2, and blocked cell proliferation [69,77]. In addition to antagonizing cell survival, miR-143 has also been shown to increase chemosensitivity to 5-FU *in vitro* [79]. miR-143 expression has been identified as an independent predictor of patient survival. Colorectal cancer patients with low levels of miR-143 expression had a significantly higher risk of having shorter cancer-specific survival and progression-free survival [78]. Taken together, the literature on let-7 and miR-143 miRNAs in colon cancer highlights the potential that miRNA-based therapies may have in targeting *KRAS*-driven colorectal cancers.

## 5. Synthetic Lethality

Another approach to targeting oncogenic *KRAS* is synthetic lethality, a phenomenon in which a combination of two or more gene mutations leads to cell death, but a mutation in one of those genes alone does not compromise cell viability [80]. The clinical significance of this approach lies in the ability to create better therapeutic options by increasing cytotoxic specificity against cancer cells without the toxicities associated with current chemotherapy. This also opens up the potential to target mutant *KRAS*-driven cancers, which thus far have no effective therapeutics. In a genome-wide screen, Luo *et al.* identified several genes in the mitotic pathway that have synthetic lethal interactions with *KRAS* [81]. In that study several potential targets for developing RNAi-based therapies or small molecule inhibitors against these synthetic lethal genes were uncovered. The complexity of synthetic lethal interactions with *KRAS* underscores the complications of regulating *KRAS* expression and activity.

## 6. Conclusion

*KRAS* mutations and their implications in cancer development have been studied for over 40 years. Colorectal cancer has the second highest prevalence of *KRAS* mutations and understanding the factors that regulate *KRAS* expression may lead to future effective therapeutic strategy. As shown with this review, *KRAS* mutations have a significant impact on colorectal cancer and the treatment strategies against it. While directly targeting *KRAS* seems to be a promising approach, *KRAS* inhibitors remain to be developed. By better understanding interactions between *KRAS* and other genes, we may then take advantage of these synthetic lethal combinations to provide additional options to treat chemotherapy-or cetuximab-refractory colorectal cancer patients harboring *KRAS* mutations. Knowledge of these synthetic lethal interactions may also enable the development of improved targeted therapies that may be more effective without the toxicities of traditional chemotherapy due to off-target killing of normal cells. Continued prospective studies and basic science research is critical in the effort to improve outcomes in colorectal cancer patients with this mutation.

## Figures and Tables

**Table 1:** Types of *KRAS* mutations.

*KRAS* mutations at codons 12 and 13 from RASCAL and RASCAL II studies				

Mutation	% of specific codon mutations		% of all codon 12/13 mutations	
		
Codon 12	RASCAL I [18]	RASCAL II [21]	RASCAL I [18]	RASCAL II [21]
G→A				
Glycine (GGT)→Serine (AGT)	7.5%	8.3%	6.8%	6.2%
Glycine (GGT)→Aspartate (GAT)	31.5%	39.3%	28.5%	39.6%
G→T				
Glycine (GGT)→Cysteine (TGT)	8.9%	10.2%	8.0%	7.7%
Glycine (GGT)→Valine (GGT)	24.2%	33.3%	21.9%	25.8%
G→C				
Glycine (GGT)→Arginine (CGT)	3.8%		3.5%	
Glycine (GGT)→Alanine (GCT)	6.2%	8.8%	5.6%	6.6%
Codon 12, unknown Point Mutation	17.8%		16.0%	
**Codon 13**				
G→A				
Glycine (GGT)→Aspartate (GAC)	83.9%	100%	14.5%	24.8%
G→T				
Glycine (GGT)→Cysteine (TGC)	6.8%		1.2%	
Glycine (GGT)→Valine (GTC)	2.1%		0.4%	
G→C				
Glycine (GGT)→Arginine (CGC)	0.7%		0.1%	
Glycine (GGT)→Alanine (GCC)	2.1%		0.4%	
Codon 13, unknown Point Mutation	5.5%		1.0%	
% of Colorectal Cancer Patients with *KRAS* mutation			37.7%	34.8%
